# Supplementary material for: Harnessing speckle for a sub-femtometre resolved broadband wavemeter and laser stabilization
Source: Nat Commun. 2017 Jun 5;8:15610. doi: 10.1038/ncomms15610 (PMC5465361; doi:10.1038/ncomms15610)
Supplement: Supplementary Information — Supplementary Tables, Supplementary Notes, Supplementary Figures and Supplementary References [file ncomms15610-s1.pdf]

Supplementary Table 1: **Summary of Integrating Sphere Wavemeter performance.**

Resolution	0.3 fm at 780 nm
Operating range	vis-nir
	(488 - 1064 nm demonstrated)
Min input power	$<$1 mW
Max acquisition rate	$>$200 kHz
Calibration stability	1.5 pm h^{-1}
Vibration dependence	not measured
Footprint (typical)	8 cm $\times$ 8 cm $\times$ 30 cm

Supplementary Table 2: **Resolution and measurement range of different interferometers, wavemeters and spectrometers.** All resolution values have been converted to femtometre at a wavelength of 780 nm if the data was available at this wavelength. For some devices [1, 2] the resolution is quoted at wavelength of 1.5 µm as the better performance was achieved in this region than for shorter wavelengths. While we have not shown the retrieval of a broad spectrum of our wavemeter and limited this assessment to narrow linewidth lasers, it is in principle possible to extend the TMM to retrieve a broad spectrum [1, 2] and therefore we compare spectrometers for completeness.

Spectrometer/wavemeter	Resolution	Measurement Range
Integrating Sphere Speckle wavemeter	0.3 fm at 780 nm	488-1064 nm
Fizeau Interferometer [3]	$<$ 2 fm at 780 nm	750-795 nm
Fabry-Perot Interferometer [4]	$<$ 2 fm	GHz at 0.3-2 µm
HighFinesse WSU2 [5]	4 fm at 420-1100 nm	330-1180 nm
Bristol Instruments[6]	22.3 fm at 780 nm	375-1100 nm
OSA high resolution [7]	40 fm	1526-1567 nm
SWIFT wavemeter [8]	40 fm	630-1000 nm
Fibre speckle spectrometer [1]	1 pm at 1.5 µm	750-795 nm
Grating Spectrometer HR [9]	8 pm	0 - 1700 nm
Tapered Fibre speckle spectrometer [2]	10 pm at 1.5 µm	500-1600 nm
µ-size Spiral Spectrometer[10]	10 pm	3 nm at 1.5 µm
OSA [11]	20 pm at 1.7 µm	600 - 1700 nm
Grating Spectrometer [12]	20 pm	200-1100 nm
µ-size Random Spectrometer[13]	75 pm	1.5-1.525 µm

Supplementary Note 1: Long-term stability

The inset of Fig. 2b of the main paper shows a short time scale of only several minutes between calibration and measurement with a precision (standard deviation) of 7.5 fm over a 3000 fm wide modulation. It is also important to evaluate the long-term stability of our speckle based wavemeter, as interference-based devices can be sensitive to changing environmental conditions [1, 2]. In previous work [1] it was found that temperature drift causes an offset in the calibration and affects the accuracy but not the precision of the reconstructed spectrum. Supplementary Figure 1a shows wavelength traces of a wavelength-modulated ECDL observed at three different times. At time $t = 10$ minutes after the calibration a standard error of 59 fm is determined in comparison to the wavemeter reference measurement. Repeating this measurement after $t = 8$ hours and $t = 19$ hours increases the standard error to 350 fm and 510 fm respectively. The error increased linearly with time which indicates that our measurement contains a systematic error, which can be corrected for. When a correction factor [1] of 1.5 pm per hour is applied to all the measurements the overall standard error decreases to 82 fm (shown in Supplementary Figure 1b) at all measurement times. The error can be further reduced by considering smaller wavelength steps in the calibration set [14].

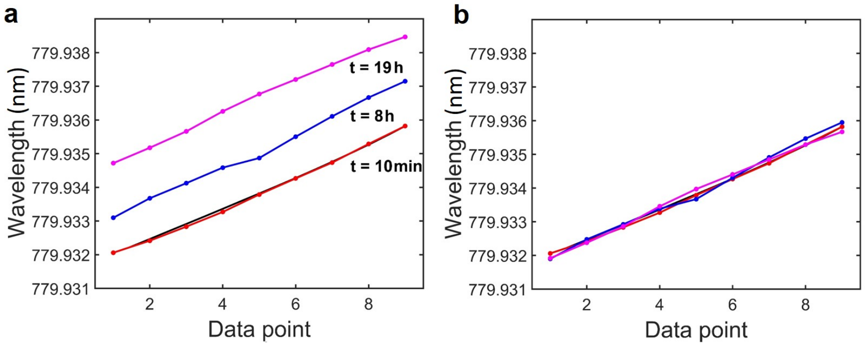

Supplementary Figure 1: **Speckle wavemeter long term stability.** (**a**) Wavelength traces of an wavelength modulated ECDL (779.932 to 779.936 nm) observed at three different times, after $t = 10$ minutes (red) with a standard deviation of 59 fm in comparison to the wavemeter reference measurement. Repeating this measurement after $t = 8$ hours (blue) and $t = 19$ hours (magenta) increases the standard error deviation to 350 fm and 510 fm. (**b**) When a correction factor of 1.5 pm per hour is applied to the measurements the standard error deviation decreases to 82 fm.

For highest precision, commercial wavelength meters usually use a frequency reference (for example a stabilized HeNe Laser) to automatically re-calibrate the device before the measurement. Our stability tests show that with an appropriate re-calibration feature the speckle wavemeter can maintain high accuracy over a long period of time. A carefully engineered system which compensates for pressure and temperature fluctuations is expected to improve the stability of the speckle wavemeter and lower the requirement on the frequency of the re-calibration.

Supplementary Note 2: Theoretical simulation

Modelling the propagation of light within the three dimensional integrating sphere to produce a speckle pattern is numerically challenging. To simplify the model we have chosen the wavelength dependent speckle pattern to be generated by the propagation of the light field through a set of diffusers spaced by the same distance as the diameter of the sphere (5 cm). In short, the diffuse reflections from the wall of the integrating sphere are approximated by a series of random phase plates and allow for a simplified calculation. The theoretical model consists of a monochromatic laser field of wavelength λ propagating along z-direction through a set of equally spaced diffusers separated by 5 cm. The number of diffusers was set to 20, which is the number of average sphere shell reflections that is described by the sphere multiplier [15]. The incident field is modelled as a Gaussian beam with spot size ω_0 and power P. Applying the paraxial wave theory [16] the evolution of the optical field $\epsilon(x, y, z)$ distorted by the refractive index variation Δn of the phase plate is calculated using the split-step beam propagation method [17]. In this system only forward scattering is of interest and the refractive index change of the phase plates are small. Importantly the diffusers are modelled as a spatially slowly varying randomly generated phase only patterns [18]. The refractive-index difference between the diffuser and the host medium is small, $\Delta n = (n_{Diffuser} - n_{Air}) = 0.001$, meaning that there is negligible backscatter and the scattering is predominantly in the forward direction. Due to the small refractive index difference, the phase plate acts in principle as random focusing elements that can be treated using scalar paraxial theory [19].

Polarization changes have effects on the generation of speckle patterns [20, 21] as orthogonal polarization components produce distinct different patterns. Within this model, only one polarization component is considered for a varying field wavelength and therefore an ideal system is considered. Including the second component would decrease the speckle contrast by $1/\sqrt{2}$ [21] and add noise but would not change the physical behaviour observed. Within this context, the paraxial approximation captures well the diffraction and interference effects that are intrinsic to the wavelength dependent generation of speckle. While propagating the field through the set of random phase plates we take into account reflecting boundaries, thus simulating a closed integrating sphere where no rays escape. An additional free space propagation (without reflecting boundary conditions) models the propagation of the distance L. The speckle intensity distribution measured by the camera is simulated by discretization of the final output field on an integer range from 0-255 to account for the limited dynamic range of the camera. The simulated speckle patterns were generated for a sinusoidally varying wavelength and then analyzed with the same program as the experimental data presented above.

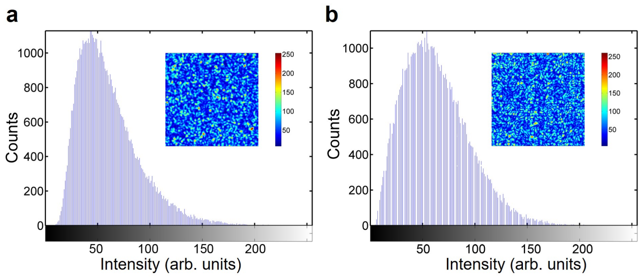

Supplementary Figure 2: **Experimental and theoretical intensity distributions.** Histogram of the intensity distribution obtained from the (**a**) integrating sphere and (**b**) the simulation. The insets show the 256 x 256 pixel speckle images in false colour, with colour bars indicating the normalized values of the intensity.

Fig. 2 shows intensity histograms of a speckle pattern generated by an integrating sphere and a speckle pattern simulated with the theoretical model. The simulations were conducted with matching experimental parameters and the speckle patterns are shown as insets. The experimental and simulated speckle pattern agree qualitatively, and indicate that the resultant complex amplitude is a circular complex Gaussian process [22, 23].

Supplementary Figure 3a-b shows results from the paraxial simulation considering a modulation amplitude equivalent to 10 fm and 100 fm respectively for 300 images (the signal period was set to 100 images). A sphere diameter of 5 cm (distance between successive random phase plates) and the free-space propagation distance from the sphere to the detector array was set to 40 cm. The simulated speckle pattern is set to a 256 × 256 element array. These results agree qualitatively well with the experimental observations from Fig. 7 where PC1 reproduces the variation while PC2 to PC4 display the second to fourth harmonic. Further simulations show that once the amplitude is increased beyond the operating range to 100 fm (Supplementary Figure 3) the signal is not correctly retrieved anymore and the reproduced signal folds in on itself as observed in the experiment (Fig. 7a). Here, the

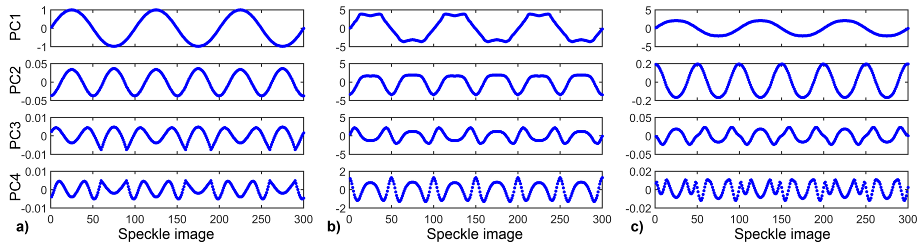

Supplementary Figure 3: **Simulated Principal Component Analysis.** (**a**) Principal components 1 to 4 for a simulated modulation amplitude of 10 fm with a sphere size D_{sphere} of 5 cm and $L = 40$ cm. The array size is 256×256 elements. (**b**) Shows a simulation as in **a** but for a larger modulation amplitude of 100 fm. Folding at the turning points is observed and the modulation is incorrectly reproduced. (**c**) 100 fm modulation for a single diffuser setup. The modulation being reproduced accurately as the variability of the speckle pattern is decreased in comparison to **b**.

variability of the speckle pattern is not captured by the finite size of the image (256×256) anymore. One can increase the range limit by enhancing the resolution or by decreasing the speckle pattern variability as for example with a single diffuser [24, 14]. We implemented this configuration in our model by simulating only one sphere reflection. Supplementary Figure 3c shows that the signal is correctly retrieved again as the variability of the speckle pattern decreased. Considering the approximate nature of this model, reasonable agreement between the experiments and simulations were achieved. The presented model captures the range limitations well and can be used to optimize future spectrometer designs.

Supplementary References

[1] Redding, B., Alam, M., Seifert, M. & Cao, H. High-resolution and broadband all-fiber spectrometers. *Optica* **1**, 175–180 (2014).

[2] Wan, N. H., *et al.*. High-resolution optical spectroscopy using multimode interference in a compact tapered fibre. *Nat. Commun.* **6**, 7762 (2015).

[3] Brachmann, J. F. S., Kinder, T. and Dieckmann, K. Calibrating an interferometric laser frequency stabilization to megahertz precision. *Appl. Opt.* **51**, 5517-5521 (2012).

[4] IS-Instruments Ultra High Resolution Spectrometer, http://www.is-instruments.com/

[5] HighFinesse wavemeter WSU2, http://www.highfinesse.com/

[6] Bristol Instruments wavemeter 671A, http://www.bristol-inst.com/

[7] High resolution OSA AP2050A http://www.apex-t.com/

[8] LW-10 Wavelength Meter: Resolution Spectra Systems http://www.resolutionspectra.com

[9] Horiba 1000M, Series II: High Resolution Research Spectrometer

[10] Redding, B., Liew, S., Bromberg, Y., Sarma, R. & Cao, H. Evanescently coupled multimode spiral spectrometer. *Optica* **3**, 956–962 (2016).

[11] Yokogawa OSA AQ6370D, http://www.yokogawa.com

[12] Ocean Optics HR4000 http://oceanoptics.com/

[13] Redding, B., Liew, S., Sarma, R. & Cao, H. Compact spectrometer based on a disordered photonic chip. *Nature Photon.* **7**, 746–751 (2013).

[14] Mazilu, M., Vettenburg, T., Di Falco, A. & Dholakia, K. Random super-prism wavelength meter. *Opt. Lett.* **39**, 96–99 (2014).

[15] Parretta, A. & Calabrese, G. About the definition of "multiplier" of an integrating sphere. *Int. J. Opt. Appl.* **3**, 119–124 (2013).

[16] Feit, M. D. & Fleck, J. A. Computation of mode properties in optical fiber waveguides by a propagating beam method. *Appl. Opt.* **19**, 1154–1164 (1980).

[17] Metzger, N. K., Wright, E. M. & Dholakia, K. Theory and simulation of the bistable behaviour of optically bound particles in the Mie size regime. *New J. Phys.* **8**, 139 (2006).

[18] Metzger, N. K., Mazilu, M., Kelemen, L., Ormos, P. & Dholakia, K. Observation and simulation of an optically driven micromotor. *J. Opt. A* **13**, 044018 (2011).

[19] Goodman, J.W. *Introduction to Fourier Optics* 3rd edn., (Mc Graw-Hill, New York, 1996).

[20] Redding, B., Popoff, S. M. & Cao, H. All-fiber spectrometer based on speckle pattern reconstruction. *Opt. Express* **21**, 6584–6600 (2013).

[21] Goodman, J.W. *Speckle Phenomena in Optics: Theory and Applications* (Roberts and Company Publishing, Englewood, 2006).

[22] Dainty, J. C. The statistics of speckle patterns, in Progress in Optics, E. Wolf (ed.), Vol. XIV, pp. 146. (1976)

[23] Webster, M. A., Webb, K. J., Weiner, A. M., Xu, J. & Cao, H. Temporal response of a random medium from speckle intensity frequency correlations. *J. Opt. Soc. Am. A* **20** 2057–2070 (2003).

[24] Kohlgraf-Owens, T. W. & Dogariu, A. Transmission matrices of random media: means for spectral polarimetric measurements. *Opt. Lett.* **13**, 2236–2238 (2010).
